# Supplementary material for: Teaching Geriatrics and Transitions of Care to Internal Medicine Resident Physicians
Source: Geriatrics (Basel). 2020 Oct 8;5(4):72. doi: 10.3390/geriatrics5040072 (PMC7720122; doi:10.3390/geriatrics5040072)
Supplement: Supplementary file 1 [file geriatrics-05-00072-s001.zip › CGEC Geriatrics Health Literacy RetroPrePostSurvey.pdf]

Date: _____

PGY: _____

Health Literacy Survey

First, please indicate how you would describe yourself **BEFORE** this rotation and ***then*** **AFTER** completing the rotation on a scale from 1 to 5 where 1 = "*Not at all*" and 5 = "*Extremely*." If you cannot comment, indicate N/A (*not applicable*) in the last column. Please circle your response.

Statement		Not at all				Extremely	N/A
Knowledgeable about definitions of health literacy	**BEFORE**	1	2	3	4	5	N/A
	**AFTER**	1	2	3	4	5	N/A
Aware of the prevalence of health literacy	**BEFORE**	1	2	3	4	5	N/A
	**AFTER**	1	2	3	4	5	N/A
Aware of effective methods for screening and identifying people with limited health literacy	**BEFORE**	1	2	3	4	5	N/A
	**AFTER**	1	2	3	4	5	N/A
Knowledgeable about laws associated with providing services to people with limited health literacy	**BEFORE**	1	2	3	4	5	N/A
	**AFTER**	1	2	3	4	5	N/A
Prepared to instruct others about health literacy (e.g. in-service)	**BEFORE**	1	2	3	4	5	N/A
	**AFTER**	1	2	3	4	5	N/A
Aware of the added health care costs associated with limited health literacy	**BEFORE**	1	2	3	4	5	N/A
	**AFTER**	1	2	3	4	5	N/A
Prepared to use "teach back" methods to assess understanding in patient-provider communication	**BEFORE**	1	2	3	4	5	N/A
	**AFTER**	1	2	3	4	5	N/A
Comfortable asking a patient to develop an action plan for their self-care	**BEFORE**	1	2	3	4	5	N/A
	**AFTER**	1	2	3	4	5	N/A
Likely to use "medication brown bag" method to assess limited health literacy	**BEFORE**	1	2	3	4	5	N/A
	**AFTER**	1	2	3	4	5	N/A
Confident in accomplishing action plan for improving geriatric care of patients with limited health literacy	**BEFORE**	1	2	3	4	5	N/A
	**AFTER**	1	2	3	4	5	N/A
Confident in ability to evaluate patient education materials appropriate for patients with limited health literacy	**BEFORE**	1	2	3	4	5	N/A
	**AFTER**	1	2	3	4	5	N/A
