# Supplementary material for: Teaching Geriatrics and Transitions of Care to Internal Medicine Resident Physicians
Source: Geriatrics (Basel). 2020 Oct 8;5(4):72. doi: 10.3390/geriatrics5040072 (PMC7720122; doi:10.3390/geriatrics5040072)

GERIATRIC ASSESSMENT

Patient Name ______________________________
Primary Care Physician ______________________ Tel/Fax: ____________________
Insurance ( ) Medicare ( ) Medi-Cal ( ) Other _____________

Consider starting your interview with Health Literacy and Hearing screening (Whisper Test).

PART I. Hospital Patient Safety

A. Delirium Screener: CAM or CAM-ICU ______ **Positive Screen >= 4/5**

Confusion Assessment Method (CAM) Worksheet (Shortened Version)

		**BOX 1**
I. ACUTE ONSET AND FLUCTUATING COURSE		
a) Is there evidence of an acute change in mental status from the patient's baseline?	No ____	Yes ____
b) Did the (abnormal) behavior fluctuate during the day, that is tend to come and go or increase and decrease in severity?	No ____	Yes ____
II. INATTENTION<br>Did the patient have difficulty focusing attention, for example, being easily distractible or having difficulty keeping track of what was being said?	No ____	Yes ____

		**BOX 2**
III. DISORGANIZED THINKING<br>Was the patient 's thinking disorganized or incoherent such as rambling or irrelevant conversation, unclear or illogical flow of ideas, or unpredictable switching from subject to subject?	No ____	Yes ____
IV. ALTERED LEVEL OF CONSCIOUSNESS<br>Overall, how would you rate the patient's level of consciousness?<br>-- Alert (normal)<br>-- Vigilant (hyperalert)<br>-- Lethargic (drowsy, easily aroused)<br>-- Stupor (difficult to arouse)<br>-- Coma (unarousable)		
Do any checks appear in this box?	No ____	Yes ____

SCORING: **If all items in Box 1 are checked and at least one item in Box 2 is checked a diagnosis of delirium is suggested.**

Adapted from Inouye SK et al, Clarifying Confusion: The Confusion Assessment Method. A New Method for Detection of Delirium. Ann Intern Med. 1990; 113:941-8. *Copyright © 2003 Hospital Elder Life Program, LLC.*

CAM-ICU Worksheet

Step 1 Level of Consciousness: RASS

Scale	Label	Description
+4	COMBATIVE	Combative, violent, immediate danger to staff
+3	VERY AGITATED	Pulls to remove tubes or catheters; aggressive
+2	AGITATED	Frequent non-purposeful movement, fights ventilator
+1	RESTLESS	Anxious, apprehensive, movements not aggressive
0	ALERT & CALM	Spontaneously pays attention to caregiver
-1	DROWSY	Not fully alert, but has sustained awakening to voice (eye opening & contact >10 sec)
-2	LIGHT SEDATION	Briefly awakens to voice (eyes open & contact <10 sec)
-3	MODERATE SEDATION	Movement or eye opening to voice (no eye contact)
-4	DEEP SEDATION	No response to voice, but movement or eye opening to physical stimulation
-5	UNAROUSEABLE	No response to voice or physical stimulation

VOICE (-1 to -3)

If RASS is ≥ -3 proceed to CAM-ICU (Is patient CAM-ICU positive or negative?)

TOUCH (-4 to -5)

If RASS is -4 or -5 → STOP (patient unconscious), RECHECK later

Sessler, et al. AJRCCM 2002;166 :1338-1344.[2]
Ely, et al. JAMA 2003; 289:2983-2991.[3]

Step 2 Content of Consciousness: CAM-ICU

Feature 1: Acute change or fluctuating course of mental status

And

Feature 2: Inattention

And

Feature 3: Altered level of consciousness

Or

Feature 4: Disorganized Thinking

Inouye, et. al. Ann Intern Med 1990; 113:941-948.[1]
Ely, et. al. CCM 2001; 29:1370-1379.[4]
Ely, et. al. JAMA 2001; 286:2703-2710.[5]

Feature 1: Acute Onset or Fluctuating Course	Score	Check here if Present
Is the pt different than his/her baseline mental status?<br>OR<br>Has the patient had any fluctuation in mental status in the past 24 hours as evidenced by fluctuation on a sedation scale (i.e., RASS), GCS, or previous delirium assessment?	Either question Yes →	☐
**Feature 2: Inattention**		
**Letters Attention Test** (See training manual for alternate **Pictures**)<br>Directions: Say to the patient, *"I am going to read you a series of 10 letters. Whenever you hear the letter 'A,' indicate by squeezing my hand."* Read letters from the following letter list in a normal tone 3 seconds apart.<br>**S A V E A H A A R T**<br>**Errors are counted when patient fails to squeeze on the letter "A" and when the patient squeezes on any letter other than "A."**	Number of Errors >2 →	☐
**Feature 3: Altered Level of Consciousness**		
Present if the Actual RASS score is anything other than alert and calm (zero)	RASS anything other than zero →	☐
**Feature 4:Disorganized Thinking**		
**Yes/No Questions** (See training manual for alternate set of questions)<br>1. Will a stone float on water?<br>2. Are there fish in the sea?<br>3. Does one pound weigh more than two pounds?<br>4. Can you use a hammer to pound a nail?<br>**Errors are counted when the patient incorrectly answers a question.**<br>**Command**<br>Say to patient: "Hold up this many fingers" (Hold 2 fingers in front of patient) "Now do the same thing with the other hand" (Do not repeat number of fingers) *If pt is unable to move both arms, for 2nd part of command ask patient to "Add one more finger"<br>**An error is counted if patient is unable to complete the entire command.**	Combined number of errors >1 →	☐
**Overall CAM-ICU**<br><br>**Feature 1 plus 2 and either 3 or 4 present = CAM-ICU positive**	**Criteria Met →**	☐ **CAM-ICU Positive (Delirium Present)**
	**Criteria Not Met →**	☐ **CAM-ICU Negative (No Delirium)**

B. Cognitive screener: Mini-Cog ____ **Positive Screen 0 – 2 (possible cognitive impairment, consider further testing, such as with MoCA or MMSE)**
Negative Screen 3 – 5

If illiterate/low education, substitute **AD8** _______ **0 – 1: Normal cognition**
≥2: Cognitive impairment likely present

or **RUDAS** _______ **< 22: abnormal**

Mini-Cog

Instructions for Administration of the Mini-Cog™

Administration	Special Instructions
1. Three Word Recall<br>Get patient's attention.<br>Say: "I am going to say three words that I want you to remember. The words are (select from word list). "Please say them for me now." If patient is unable to repeat after 3 tries, then go to clock drawing test.	The following word lists have been used in one or more clinical studies:[1–3]<br>- ***Version 1***: • Banana • Sunrise • Chair<br>- ***Version 2***: • Daughter • Heaven • Mountain<br>- ***Version 3***: • Village • Kitchen • Baby<br>- ***Version 4***: • River • Nation • Finger<br>- ***Version 5***: • Captain • Garden • Picture<br>- ***Version 6***: • Leader • Season • Table
2. Clock Drawing Test (CDT)<br>Say in order: "Please draw a clock. Start by drawing a large circle." (when done, say) "Put all the numbers in the circle." (when done, say) "Now set the hands to show 11:10 (10 past 11) OR 8:20 OR 1:45.	• A clock should not be visible to the patient during this task.<br>• Use either a blank piece of paper and have patient draw circle OR provide a preprinted circle – administration would then be to ask the patient to put in all the numbers like the face of a clock.<br>• Repeat instructions as needed. This is not a memory test. Move to next step if clock is not complete within 3 minutes.<br>• Inability or refusal to draw a clock is scored abnormal (0 points).
3. Say: "What were the three words I asked you to remember?	Ask the patient to recall the three words you stated in Step 1.

Scoring

Word recall ___ (0-3 points)	1 point for each word spontaneously recalled without cueing.
Clock draw ___ (0 or 2 points)	• Normal clock = 2 points. A normal clock has all numbers placed in the correct sequence and approximately correct position (eg, with 12, 3, 6, and 9 in anchor positions) with no missing or duplicate numbers. Hands are pointing to the 11 and 2 (11:10) or the 8 and 4 (8:20) or 1 and 9 (1:45). (Length of hands less important).<br>• Abnormal clock = 0 points.
Total = _______ (0- 5 points)	Total score = word recall score + clock score<br>Negative screen for cognitive impairment: Mini-Cog™ 4-5 score<br>Positive screen for cognitive impairment: Mini-Cog™ 0-3 score

References/Copyright Information

1. Borson S, Scanlan J, Brush M, Vitaliano P, Dokmak A. The mini-cog: a cognitive "vital signs" measure for dementia screening in multi-lingual elderly. *Int J Geriatr Psychiatry*. 2000;15(11):1021–1027. 2. Borson S, Scanlan JM, Chen P, Ganguli M. The Mini-Cog as a screen for dementia: validation in a population-based sample. *J Am Geriatr Soc*. 2003;51(10):1451–1454. 3. McCarten JR, Anderson P, Kuskowski MA, et al. Finding dementia in primary care: the results of a clinical demonstration project. *J Am Geriatr Soc*. 2012;60(2):210–217.

Mini-Cog™ Copyright S. Borson. All rights reserved. Used with permission of the author in educational and clinical materials developed by the Alzheimer's Association.

Clock Drawing Test

Patient Name: ______________________________ Date: ______________

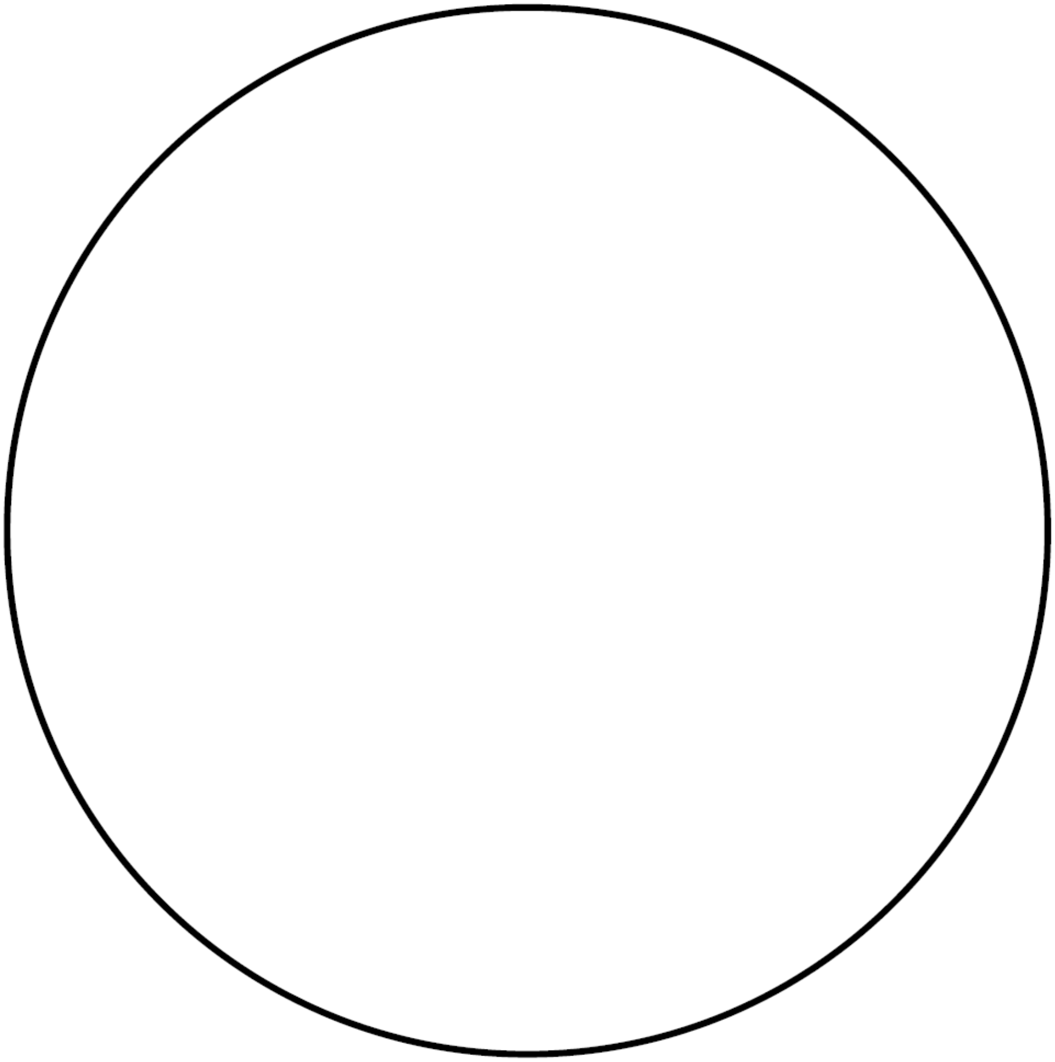

If patient has low education or is illiterate, use the AD8 to interview an informant (such as by calling a caregiver or family member). If the AD8 is positive, evaluate with the MoCA-BLIND.

AD8 Dementia Screening Interview

Patient ID#:__________
CS ID#:__________
Date:__________

Remember, "Yes, a change" indicates that there has been a change in the last several years caused by cognitive (thinking and memory) problems.	**YES, A change**	**NO, No change**	**N/A, Don't know**
**1.** Problems with judgment (e.g., problems making decisions, bad financial decisions, problems with thinking)			
**2.** Less interest in hobbies/activities			
**3.** Repeats the same things over and over (questions, stories, or statements)			
**4.** Trouble learning how to use a tool, appliance, or gadget (e.g., VCR, computer, microwave, remote control)			
**5.** Forgets correct month or year			
**6.** Trouble handling complicated financial affairs (e.g., balancing checkbook, income taxes, paying bills)			
**7.** Trouble remembering appointments			
**8.** Daily problems with thinking and/or memory			
**TOTAL AD8 SCORE**			

Adapted from Galvin JE et al, The AD8, a brief informant interview to detect dementia, Neurology 2005:65:559-564

Copyright 2005. The AD8 is a copyrighted instrument of the Alzheimer's Disease Research Center, Washington University, St. Louis, Missouri. All Rights Reserved.

C. Age & Weight Adjusted GFR:

Estimated GFR (creatinine clearance) by Cockcroft-Gault: ___________

Cockcroft-Gault: With aging, proportion of muscle mass to fat decreases. Creatinine clearance is less accurate for predicting GFR. The Cockcroft-Gault equation adjusts for age and weight:

CrCl = (140-age) * (Wt in kg) * (0.85 if female) / (72 * Cr)

Online calculator further adjusts weight if under- or overweight**:**
http://www.mdcalc.com/creatinine-clearance-cockcroft-gault-equation/

D. Medication Safety: Review all current medications and list those with potential age-related side effects and recommendations. Reference: American Geriatrics Society Beers Criteria for Potentially Inappropriate Medication Use in Older Adults.

Age-related Medication Issues ____________________________________

Adverse Reactions

Medication Name	Potential Adverse Reaction	Recommendation

Potential Important Drug Interactions:

Medication 1	Medication 2	Drug Interaction

Medications to be renally-dosed based on eGFR above:

____

____

E. Incontinence:

Urinary incontinence	☐ No	☐ Yes, if new, related to acute illness (e.g., UTI) or medications (e.g. diuretic)
Foley catheter in place	☐ No	☐ Yes, what is medical indication or patient preference? If none, D/C Foley.
Bowel incontinence	☐ No	☐ Yes

F. Skin Evaluation:

Review nursing assessment:

Location	Size	Stage	Drainage

If pressure ulcer(s) present or if bedbound/poor mobility or delirious, recommend “turn patient q 2 hours.”

G. History of Falls: ☐ No ☐ Yes

PART II. Mobility/Falls

A. Assistive device Uses a walking aid such as a cane or a walker?

☐ No ☐ Yes, **which ones?**

☐ Cane ☐ Walker ☐ Wheelchair

B. Fear of falling ☐ No ☐ Yes (increases risk of falls)

C. Fallen in past year? ☐ No ☐ Yes, **circumstances of the fall(s):**

Tripped over something	☐ Yes ☐ No
Lightheadedness or palpitations prior?	☐ Yes ☐ No
Loss of consciousness?	☐ Yes ☐ No
Injury or needed to see a doctor?	☐ Yes ☐ No
Able to get up by self?	☐ Yes ☐ No
Vision impairment	☐ Yes ☐ No
Cognitive impairment	☐ Yes ☐ No

D. Physical Exam:

Vision problems ☐ No ☐ Yes

Hearing Evaluation:

Whisper Test: Stand to the side and slightly behind the patient at arms length. Have the patient look forward and cover the opposite ear. Whisper "1, 2, 3," ask the patient if they heard you and to please repeat what you said. Next, stand on the other side of the patient. This time whisper, "A, B, C," ask the patient if they heard you and to please repeat what you said.

Left Ear: Did the patient hear you? Yes/No Right Ear: Did the patient hear you? Yes/No

Orthostatic Blood Pressure _____/____ Lying, pulse ___ ; _____/____ Standing, pulse ___

Gait: ☐ Normal ☐ Abnormal ☐ Unable due to medical condition

Abnormal if:

☐ Hesitant start ☐ Extended arms ☐ Broad-based gait ☐ Path deviates

☐ Heels do not clear floor (shuffles) ☐ Heels do not clear toes of other foot

Balance

Side-by-side stance, stable 10 sec	☐ No	☐ Yes
Semi-tandem stance, stable 10 sec	☐ No	☐ Yes
Full tandem, stable 10 sec	☐ No	☐ Yes

Neuromuscular

Quad strength (chair rise)	☐ No	☐ Yes (one attempt to rise)
Normal hip range of motion	☐ No	☐ Yes
Rigidity (e.g., cogwheeling)	☐ No	☐ Yes, ☐ Stroke?
Bradykinesia	☐ No	☐ Yes, ☐ Parkinsonism?
Tremor	☐ No	☐ Yes, ☐ Parkinsonism?

PART III. Function

Patients who are dependent on others for activities of daily living have poorer life expectancy. The patient's functional status helps determine safe discharge planning.

A. Index of Independence in Activities of Daily Living (Basic ADLs) ________ **/ 6**

**ACTIVITIES**<br>POINTS (1 OR 0)	**INDEPENDENCE:**<br>(1 POINT)<br>**NO** supervision, direction or personal assistance	**DEPENDENCE:**<br>(0 POINTS)<br>**WITH** supervision, direction, personal assistance or total care
**BATHING**<br>POINTS:___________	**(1 POINT)** Bathes self completely or needs help in bathing only a single part of the body such as the back, genital area or disabled extremity.	**(0 POINTS)** Needs help with bathing more than one part of the body, getting in or out of the tub or shower. Requires total bathing.
**DRESSING**<br>POINTS:___________	**(1 POINT)** Gets clothes from closets and drawers and puts on clothes and outer garments complete with fasteners. May have help tying shoes.	**(0 POINTS)** Needs help with dressing self or needs to be completely dressed.
**TOILETING**<br>POINTS:___________	**(1 POINT)** Goes to toilet, gets on and off, arranges clothes, cleans genital area without help.	**(0 POINTS)** Needs help transferring to the toilet, cleaning self or uses bedpan or commode.
**TRANSFERRING**<br>POINTS:___________	**(1 POINT)** Moves in and out of bed or chair unassisted. Mechanical transferring aides are acceptable.	**(0 POINTS)** Needs help in moving from bed to chair or requires a complete transfer.
**CONTINENCE**<br>POINTS:___________	**(1 POINT)** Exercises complete self control over urination and defecation.	**(0 POINTS)** Is partially or totally incontinent of bowel or bladder.
**FEEDING**<br>POINTS:___________	**(1 POINT)** Gets food from plate into mouth without help. Preparation of food may be done by another person.	**(0 POINTS)** Needs partial or total help with feeding or requires parenteral feeding.

TOTAL POINTS = ______ 6 = High (*patient independent*) 0 = Low (*patient very dependent*)

Slightly adapted from Katz, S., Down, T.D., Cash, H.R., & Grotz, R.C. (1970) Progress in the development of the index of ADL. *The Gerontologist, 10*(1), 20-30.

Copyright © The Gerontological Society of America. Reproduced [Adapted] by permission of the publisher.

B. Instrumental Activities of Daily Living Scale (IADLs) ________ / 8

A. Ability to Use Telephone

1. Operates telephone on own initiative; looks up and dials numbers.. 1
2. Dials a few well-known numbers................................ 1
3. Answers telephone, but does not dial......................... 1
4. Does not use telephone at all...................................... 0

B. Shopping

1. Takes care of all shopping needs independently........ 1
2. Shops independently for small purchases.................. 0
3. Needs to be accompanied on any shopping trip 0
4. Completely unable to shop 0

C. Food Preparation

1. Plans, prepares, and serves adequate meals independently.. 1
2. Prepares adequate meals if supplied with ingredients... 0
3. Heats and serves prepared meals or prepares meals but does not maintain adequate diet.......................... 0
4. Needs to have meals prepared and served.................. 0

D. Housekeeping

1. Maintains house alone with occasion assistance (heavy work)... 1
2. Performs light daily tasks such as dishwashing, bed making.. 1
3. Performs light daily tasks, but cannot maintain acceptable level of cleanliness 1
4. Needs help with all home maintenance tasks............ 1
5. Does not participate in any housekeeping tasks........ 0

E. Laundry

1. Does personal laundry completely 1
2. Launders small items, rinses socks, stockings, etc........ 1
3. All laundry must be done by others 0

F. Mode of Transportation

1. Travels independently on public transportation or drives own car.. 1
2. Arranges own travel via taxi, but does not otherwise use public transportation 1
3. Travels on public transportation when assisted or accompanied by another 1
4. Travel limited to taxi or automobile with assistance of another.. 0
5. Does not travel at all 0

G. Responsibility for Own Medications

1. Is responsible for taking medication in correct dosages at correct time.. 1
2. Takes responsibility if medication is prepared in advance in separate dosages.................................... 0
3. Is not capable of dispensing own medication 0

H. Ability to Handle Finances

1. Manages financial matters independently (budgets, writes checks, pays rent and bills, goes to bank); collects and keeps track of income............................. 1
2. Manages day-to-day purchases, but needs help with banking, major purchases, etc 1
3. Incapable of handling money 0

Scoring: For each category, circle the item description that most closely resembles the client's highest functional level (either 0 or 1).

Lawton, M.P., & Brody, E.M. (1969). Assessment of older people: Self-maintaining and instrumental activities of daily living. *The Gerontologist*, *9*(3), 179-186.

C. Medication Management ☐ Self ☐ Other ____________________

D. Life Expectancy

☐ < 6 mo ☐ < 1 - 5 years ☐ <10 years ☐ >10 years (consider cancer screening)

Estimating life expectancy helps patients and caregivers make informed choices regarding health care. Life tables can be used or an online calculator based on clinical setting is available at http://eProgonosis.ucsf.edu.

PART IV. Psychosocial & Advance Care Planning

A. Depression Screener: **PHQ-2** _____ **>0 is a positive screen, complete full PHQ-9**
PHQ-9 ______ **Depression Severity** _______

Interpretation of Total Score

Total Score	Depression Severity
1-4	Minimal depression
5-9	Mild depression
10-14	Moderate depression
15-19	Moderately severe depression
20-27	Severe depression

Is patient delirious? ☐ No ☐ Yes, do not administer depression screening

Patient Health Questionnaire (PHQ-2)

Initial screen is for the two major criteria for depression: anhedonia and depressed mood.

Over the last *2 weeks*, how often have you been bothered by any of the following problems? *(use "✓" to indicate your answer)*	Not at all	Several days	More than half the days	Nearly every day
**1.** Little interest or pleasure in doing things	0	1	2	3
**2.** Feeling down, depressed, or hopeless	0	1	2	3

PHQ-2: add columns: ___________________________

Negative Screen = 0
Positive Screen >= 1, complete remainder of PHQ-9 (all 9 criteria for depression)

Over the last *2 weeks*, how often have you been bothered by any of the following problems?
(use "✓" to indicate your answer)

	Not at all	Several days	More than half the days	Nearly every day
**1.** Little interest or pleasure in doing things	0	1	2	3
**2.** Feeling down, depressed, or hopeless	0	1	2	3
**3.** Trouble falling or staying asleep, or sleeping too much	0	1	2	3
**4.** Feeling tired or having little energy	0	1	2	3
**5.** Poor appetite or overeating	0	1	2	3
**6.** Feeling bad about yourself—or that you are a failure or have let yourself or your family down	0	1	2	3
**7.** Trouble concentrating on things, such as reading the newspaper or watching television	0	1	2	3
**8.** Moving or speaking so slowly that other people could have noticed. Or the opposite—being so figety or restless that you have been moving around a lot more than usual	0	1	2	3
**9.** Thoughts that you would be better off dead, or of hurting yourself	0	1	2	3

add columns ______ + ______ + ______

(Healthcare professional: For interpretation of TOTAL, please refer to accompanying scoring card). TOTAL: ______

10. If you checked off *any problems*, how *difficult* have these problems made it for you to do your work, take care of things at home, or get along with other people?

Not difficult at all ______
Somewhat difficult ______
Very difficult ______
Extremely difficult ______

B. Advance Care Planning:

Who is patient's Spokesperson? ☐ Self ☐ Other ___________________ Relationship __________
Contact #_______________
☐ Caregiver ____

Does the patient have a Medical Durable Power of Attorney for Health Care or an Advance Directive?
Yes/No _____________

Preferences: ____
☐ POLST ____________________ ☐ DNR/DNI

Document your Advance Care Planning Discussion in ORCHID, select Ad Hoc: Ambulatory: Advance Care Planning (ACP) and save to chart by clicking the "check mark" button.

Health Literacy: ______ Ask: "How confident are you filling out medical forms by yourself?" OR "How often do you need to have someone help you when you read instructions, pamphlets, or other written material from your doctor or pharmacy?" If patient needs help, positive screen for low health literacy. If education > high school, consider administering Newest Vital Sign screening tool.

Spiritual assistance request: ☐ No ☐ Yes

May patient benefit from referral to community services? ☐ Yes: place eConsult to the Geriatrics Navigator

PART V. Schematic of Assessment Domains

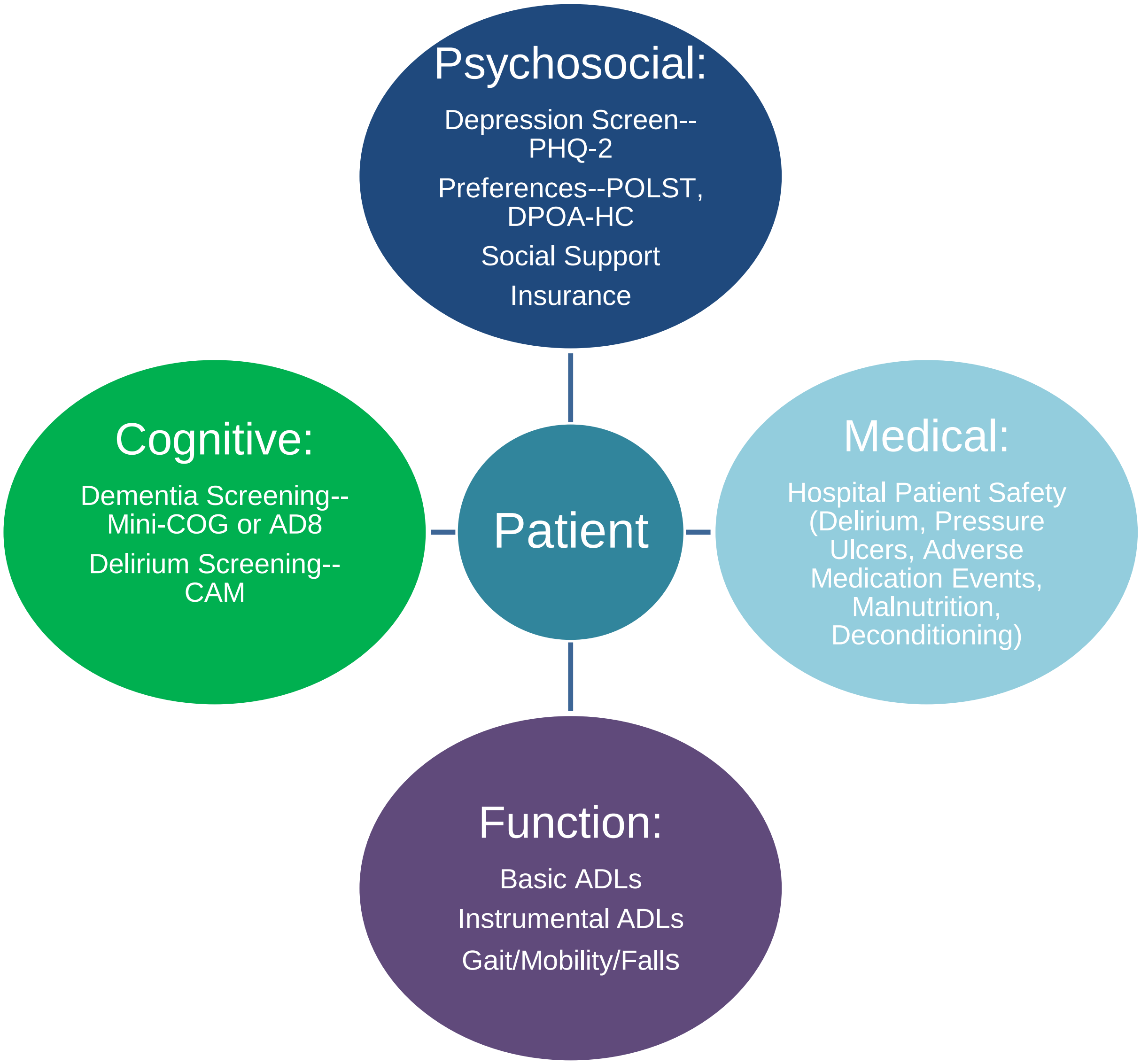

Supplement: Supplementary file 1 [file geriatrics-05-00072-s001.zip › Geriatric Consultation Assessment Packet.pdf]
