# Supplementary material for: Teaching Geriatrics and Transitions of Care to Internal Medicine Resident Physicians
Source: Geriatrics (Basel). 2020 Oct 8;5(4):72. doi: 10.3390/geriatrics5040072 (PMC7720122; doi:10.3390/geriatrics5040072)
Supplement: Supplementary file 1 [file geriatrics-05-00072-s001.zip › Geriatrics and Transitions of Care Post-Rotation Survey.pdf]

Geriatrics and Transitions of Care Post-Rotation Survey

Date: ____________ PGY level: ____________

Instructions: Please indicate your opinion about each of the statements by circling the appropriate response at the right of the statement. There are no right or wrong answers. We are interested in your frank opinions. Your responses are confidential.

1. The first set of questions refers to the quality of each clinical experience. Was the experience helpful in achieving the course objectives of learning about the care of geriatric patients?

Clinical Experience	Not helpful at all	Minimally helpful	Somewhat helpful	Very helpful	Not Applic-able
a) Geriatric Inpatient Consults (overall)	1	2	3	4	NA
b) Surgical Automatic Consults	1	2	3	4	NA
c) Requested consults (Surgical, Medical, other)	1	2	3	4	NA
d) ICU consults	1	2	3	4	NA
e) Ward consults	1	2	3	4	NA
f) Transitions of Care Clinic	1	2	3	4	NA
g) Transitions of Care Activities (e.g., contacting PCP, sending discharge information to other providers)	1	2	3	4	NA

2. I received feedback during this rotation: ☐ YES ☐ NO

	Not helpful at all	Minimally helpful	Somewhat helpful	Very helpful	Not Applic-able
The feedback I received was (CIRCLE)	1	2	3	4	NA

3. Please rate how confident you are in your ability to successfully accomplish each of the following tasks. Indicate your degree of confidence, from 1 (No Confidence At All) to 5 (Completely Confident). Please circle your answer.

I am confident in my ability to:	Before the Geriatrics Rotation	After Completion of the Geriatrics Rotation
Perform a functional status evaluation which includes assessment of ADLs and IADLs	1 2 3 4 5	1 2 3 4 5

I am confident in my ability to:	Before the Geriatrics Rotation					After Completion of the Geriatrics Rotation				
Diagnose and initially manage delirium	1	2	3	4	5	1	2	3	4	5
Diagnose and initially manage dementia	1	2	3	4	5	1	2	3	4	5
Diagnose and initially manage acute pain in hospitalized patients	1	2	3	4	5	1	2	3	4	5
Diagnose and initially manage falls and gait problems	1	2	3	4	5	1	2	3	4	5
Initially manage osteoporotic fractures as a medicine consultant	1	2	3	4	5	1	2	3	4	5
Practice optimal geriatric pharmacotherapy (understanding drug related problems in the elderly and reducing inappropriate prescribing)	1	2	3	4	5	1	2	3	4	5
Discuss advance care planning with patients and families	1	2	3	4	5	1	2	3	4	5
Understand the role of other practice settings (long-term care, hospice, SNF) including indications, basic regulatory issues, and differences from acute care settings	1	2	3	4	5	1	2	3	4	5
Determine optimal placement of patient at discharge based on functional needs and insurance resources	1	2	3	4	5	1	2	3	4	5
Work effectively within a multidisciplinary team and make appropriate referrals to social workers, discharge planners, pharmacists, rehabilitation therapists and dietitians	1	2	3	4	5	1	2	3	4	5
Communicate effectively with outpatient providers about patient's hospitalization and recommended follow-up	1	2	3	4	5	1	2	3	4	5

Please comment on any of the above questions: ___________________________

____

____

4. Overall Geriatrics Rotation Evaluation
Please circle your answer.

	**Poor**	**Below Average**	**Average**	**Above Average**	**Excellent**	**N/A**
Overall quality of rotation	1	2	3	4	5	NA
Met learning objectives	1	2	3	4	5	NA
Relevance of topics	1	2	3	4	5	NA
Quality of clinical cases	1	2	3	4	5	NA
Quality of didactic sessions	1	2	3	4	5	NA
Quality of teaching materials	1	2	3	4	5	NA
Quality of bedside teaching	1	2	3	4	5	NA
Quality of clinical skills observation feedback	1	2	3	4	5	NA
Opportunities for discussion	1	2	3	4	5	NA
Facilities	1	2	3	4	5	NA
Staff Support	1	2	3	4	5	NA
Met overall expectations	1	2	3	4	5	NA

General Comments (e.g., highlights of the rotation, handout/audiovisual use; opportunity for questions; topic coverage; relevance to my needs, etc.):

____

____

____

Please suggest specific ways in which this rotation could be improved:

____

____

____
