# Supplementary material for: Teaching Geriatrics and Transitions of Care to Internal Medicine Resident Physicians
Source: Geriatrics (Basel). 2020 Oct 8;5(4):72. doi: 10.3390/geriatrics5040072 (PMC7720122; doi:10.3390/geriatrics5040072)
Supplement: Supplementary file 1 [file geriatrics-05-00072-s001.zip › Geriatrics Communications Skills Mini-CEX.pdf]

Geriatrics Communication Skills Mini-CEX

Name: ________________________ **PGY level:** ____________ ☐ **Pre-test** ☐ **Post-Test**

Evaluator: ________________________________ **Date:** _____________________

1. Adapts appropriately to communication barriers with patient such as sensory impairment (hearing loss) and language barrier (staff or phone interpreter).

1	2	3	4	5	6	7	8	9
	UNSATISFACTORY			SATISFACTORY			SUPERIOR	

Comments:

2. Screens for low health literacy (e.g., ask about formal education, if < high school, ask about print literacy, if > high school, use validated screening tool).

1	2	3	4	5	6	7	8	9
	UNSATISFACTORY			SATISFACTORY			SUPERIOR	

Comments:

3. Solicits patient's and/or caregivers' understanding of disease/situation.

1	2	3	4	5	6	7	8	9
	UNSATISFACTORY			SATISFACTORY			SUPERIOR	

Comments:

4. Uses Teachback Method appropriately (Asks pt to explain concept/change in nonjudgmental manner; If corrections needed, modifies explanation; Elicits repeat teach-back from patient).

1	2	3	4	5	6	7	8	9
	UNSATISFACTORY			SATISFACTORY			SUPERIOR	

Comments:

5. Uses appropriate language for patient's level of understanding and literacy.

1	2	3	4	5	6	7	8	9
	UNSATISFACTORY			SATISFACTORY			SUPERIOR	

Comments:

6. Avoids medical jargon.

1	2	3	4	5	6	7	8	9
	UNSATISFACTORY			SATISFACTORY			SUPERIOR	

Comments:

7. Gives patient opportunity/time to talk.

1	2	3	4	5	6	7	8	9
	UNSATISFACTORY			SATISFACTORY			SUPERIOR	

Comments:

8. Demonstrates active listening (e.g., eye contact, undivided attention to patient, reflects back what patient states).

1	2	3	4	5	6	7	8	9
	UNSATISFACTORY			SATISFACTORY			SUPERIOR	

Comments:

9. Solicits patient and/or caregiver preferences on decision-making on plan of care.

1	2	3	4	5	6	7	8	9
	UNSATISFACTORY			SATISFACTORY			SUPERIOR	

Comments:

10. Treats patient and caregiver with respect and compassion.

1	2	3	4	5	6	7	8	9
	UNSATISFACTORY			SATISFACTORY			SUPERIOR	

Comments:

Comments: ____

____

____

____

This trainee has been observed and been given feedback.

____

TRAINEE SIGNATURE

____

FACULTY SIGNATURE
