# Supplementary material for: Teaching Geriatrics and Transitions of Care to Internal Medicine Resident Physicians
Source: Geriatrics (Basel). 2020 Oct 8;5(4):72. doi: 10.3390/geriatrics5040072 (PMC7720122; doi:10.3390/geriatrics5040072)
Supplement: Supplementary file 1 [file geriatrics-05-00072-s001.zip › Geriatrics Skills Checklist.pdf]

Geriatrics Skills Checklist

Trainee Name: ________________________________ **PGY**_________

Rotation Name and Dates: ____

	Date	Date	Constructive Feedback for Improvement	Attg Initial
**Hospitalized Patients**				
Delirium screening tool (CAM, CAM-ICU)				
Dementia screening tool (Mini-Cog, MMSE, MoCA)				
Functional assessment Score (Katz ADLs and Lawton IADLs)				
Health Literacy screening tool (Newest Vital Sign)				
Cockcroft-Gault Equation to calculate GFR (Online MedCalc)				
**Transitions of Care Clinic**				
Depression screening tool (PHQ-2, PHQ-9)				
Gait assessment (Tinetti or Timed Up and Go Test)				
Medication Reconciliation post-discharge (Patient Health Record)				
Teachback Method for assessing patient understanding (paired with **mini-CEX**)				
Estimate Prognosis <5 years, 5-10 years, or >10 years (EPrognosis or Life Expectancy Table)				

Tools can be used in either setting if appropriate to the particular patient case.
