# Supplementary material for: Teaching Geriatrics and Transitions of Care to Internal Medicine Resident Physicians
Source: Geriatrics (Basel). 2020 Oct 8;5(4):72. doi: 10.3390/geriatrics5040072 (PMC7720122; doi:10.3390/geriatrics5040072)
Supplement: Supplementary file 1 [file geriatrics-05-00072-s001.zip › Personal Health Record.pdf]

Personal Health Record

Health Services
LOS ANGELES COUNTY

This Personal Health Record belongs to:

____

Primary physician:

_________________________ (____) - _____-_____
primary physician's name *phone number*

Specialty physician(s):

_________________________ (____) - _____-_____
physician name/specialty *phone number*

_________________________ (____) - _____-_____
physician name/specialty *phone number*

Pharmacy:

_________________________ (____) - _____-_____
pharmacy name *phone number*

Remember to take this Personal Health Record with you to all of your hospital and doctor visits.

I am receiving transitional care services from:

_____________________________ (_____) - _____-_____
name of facility *phone number*

My coach is:

____
name of transition coach

(_____) - _____-_____ (_____) - _____-_____
office phone number *alternate phone number*

I am receiving home care from:

_____________________________ (_____) - _____-_____
Name of home health agency *phone number*

Other community services I am receiving:

_____________________________ (_____) - _____-_____
name of service *phone number*

_____________________________ (_____) - _____-_____
name of service *phone number*

_____________________________ (_____) - _____-_____
name of service *phone number*

PERSONAL INFORMATION

My home address is:

____
number and street

______________________________ _________
city and state *zip code*

My contact telephone numbers are:

(_____) - _____-_____ (_____) - _____-_____
home phone number *alternate phone number*

Advance Directive(s) (Check all that apply.)

☐ Living Trust ☐ DPAHC ☐ POLST ☐ DNR

_____________________________ (____) - _____-_______
Name of Health Care Proxy *Phone Number*

Location of my Advance Directive(s)

____

Organ Donor ☐ Yes ☐ **N**o

CAREGIVER INFORMATION

My primary caregiver is:

(______) - _______-_________ (______) - ______-__________
home phone number *alternate phone number*

____
relationship

RECORD OF HOSPITALIZATIONS:

Admitted ___/___/_____ Discharged___/___/_____

Name of Hospital: _____________________________

Reason for Hospitalization:

____

Admitted ___/___/_____ Discharged___/___/_____

Name of Hospital: _____________________________

Reason for Hospitalization:

____

Admitted ___/___/_____ Discharged___/___/_____

Name of Hospital: _____________________________

Reason for Hospitalization:

____

MY PERSONAL HEALTH GOAL(S):

____

____

____

____

____

____

MY HEALTH CONDITIONS:

1. ____

Red Flag:___________________________________

Action Step:_________________________________

2. ____

Red Flag:___________________________________

Action Step:_________________________________

3. ____

Red Flag:___________________________________

Action Step:_________________________________

FOLLOW-UP PHYSICIAN APPOINTMENT(S):

Appointment date ___/___/_____ Time: _____

Name of primary care physician: ______________________

Appointment date ___/___/_____ Time: _____

Name of specialty physician/clinic_____________________

Appointment date ___/___/_____ Time: _____

Name of specialty physician/clinic_____________________

APPOINTMENT FOR BLOOD TEST(S)

Appointment date ___/___/_____ Time: _____

Appointment date ___/___/_____ Time: _____

Appointment date ___/___/_____ Time: _____

APPOINTMENT FOR SPECIAL STUDIES

Appointment date ___/___/_____ Time: _____

Type of study : _____________________________________

Appointment date ___/___/_____ Time: _____

Type of study : _____________________________________

Appointment date ___/___/_____ Time: _____

Type of study : _____________________________________

QUESTIONS FOR MY PHYSICIAN(S)

1. ______________________________

____

____

____

2. ______________________________

____

____

____

3. ______________________________

____

____

____

4. ______________________________

____

____

RECORD OF MY MEDICINES

Allergies: ____________________________________

Part D Plan: _________________________________

MY MORNING MEDICINES				
**Name and dose of medicine**	**How many**	**Reason**	**New** *Yes or No*	**Place Pill here**

Please include Over-the-Counter and Herb preparations

MY AFTERNOON MEDICINES				
Name and dose of medicine	How many	Reason	New *Yes or No*	Place Pill here

Please include Over-the-Counter and Herb preparations

MY EVENING MEDICINES				
**Name and dose of medicine**	**How many**	**Reason**	**New** *Yes or No*	**Place Pill here**

Please include Over-the-Counter and Herb preparations

MY BEDTIME MEDICINES				
**Name and dose of medicine**	**How many**	**Reason**	**New** *Yes or No*	**Place Pill here**

Please include Over-the-Counter and Herb preparations

MY DISCHARGE PREPARATION CHECKLIST

Before I leave the hospital, I accept the responsibility to:

1. Be involved in decisions about what will take place after I leave the hospital.

2. Get answers to my most important questions prior to leaving the hospital.

3. Let my family or someone close to me knows that I am coming home, what I will need (medicine, supplies, and medical equipment), and what I need help with once I leave the hospital.

4. If I am going directly home, schedule a follow-up appointment with my doctor, and I have transportation to this appointment.

5. Review my discharge instructions and ask for help if I don't understand them.

This material was adapted from a tool developed by Dr. Eric Coleman, UCHSC, HCPR, with funding from the John A. Hartford Foundation and the Robert Wood Johnson Foundation

MY DISCHARGE PREPARATION CHECKLIST

Before I leave the hospital, I need to know:

1. Where I am going after I leave the hospital and what will happen to me once I arrive.

2. The name and phone number of a person I should contact if a problem arises during my transfer.

3. What my medicines are, how I will obtain them today, and how to take them.

4. The potential side effects of my medicines and whom I should call if I experience them.

5. What symptoms I need to watch out for and whom to call should I notice them.

6. How to keep my health problems from becoming worse.

7. When my next medical appointment is scheduled.

This material was adapted from a tool developed by Dr. Eric Coleman, UCHSC, HCPR, with funding from the John A. Hartford Foundation and the Robert Wood Johnson Foundation

NOTES:
