# Supplementary material for: Teaching Geriatrics and Transitions of Care to Internal Medicine Resident Physicians
Source: Geriatrics (Basel). 2020 Oct 8;5(4):72. doi: 10.3390/geriatrics5040072 (PMC7720122; doi:10.3390/geriatrics5040072)
Supplement: Supplementary file 1 [file geriatrics-05-00072-s001.zip › UCLA Attitudes scale with key.pdf]

DIRECTIONS: Please use the scale to indicate the degree to which you agree or disagree with each statement. There are no right or wrong answers. The best response is the one that truly reflects your personal opinion. "Old people" and "elderly patients" mentioned in the questions refer to persons aged 65 or older.

		Strongly Disagree	Somewhat Disagree	Neutral	Somewhat Agree	Strongly Agree
1.	Most old people are pleasant to be with.	1	2	3	4	5
2.	The federal government should reallocate money from Medicare to research on AIDS or pediatric diseases.	1	2	3	4	5
3.	If I have the choice, I would rather take care of younger people than elderly ones.	1	2	3	4	5
4.	It is society's responsibility to provide care for its elderly persons.	1	2	3	4	5
5.	Medical care for old people uses up too much human and material resources.	1	2	3	4	5
6.	As people grow older, they become less organized and more confused.	1	2	3	4	5
7.	Elderly patients are more appreciative of the care they receive than are younger ones.	1	2	3	4	5
8.	I think taking a medical history from elderly patients is frequently an ordeal.	1	2	3	4	5
9.	I tend to have more sympathy towards elderly patients than younger ones.	1	2	3	4	5
10.	Old people in general do not contribute much to society.	1	2	3	4	5
11.	Treatment of chronically ill old patients is hopeless.	1	2	3	4	5
12.	Old persons don't contribute their fair share towards paying for their health care.	1	2	3	4	5
13.	In general, old people act too slow for modern society.	1	2	3	4	5
14.	It is interesting listening to old people's accounts of their past experiences.	1	2	3	4	5

KEY

Item	Sign
1	+
2	-
3	-
4	+
5	-
6	-
7	+
8	-
9	+
10	-
11	-
12	-
13	-
14	+

For Scoring, you need to reverse (i.e., 1 to 5, 2 to 4, 3 stays, 4 to 2, and 5 to 1) the scores on the negatively worded items before you add them to the scores on the positively worded items to obtain a total score.
